# A Treatise on the Practice of Medicine

**Published:** 1887-02

**Authors:** 


### Reviews.

**A Treatise on the Practice of Medicine** for the use of Students and Practitioners of Medicine. By ROBERTS BARTHOLOW, M. D., L. L. D., Prof. Materia Medica and Therapeutics, Jefferson Medical College, etc., etc. Sixth edition, revised and enlarged. New York: D. Appleton & Co. 1886.

Bartholow's "Practice" is so well known and so generally esteemed that any criticism on the work itself is unneccssary. Indeed a book which reaches a sixth edition within six years needs no commendation. In this last edition some new chapters have been introduced, but the amount of new material is not large. Those who have the fifth edition will not find it necessary to purchase this one. Those who have not, can buy this last edition with the assurance that they will obtain the latest information upon any subject within its scope. The value of the work lies in its eminently practical character.

